# Spontaneous ruptured splenic artery aneurysm: a case report

**DOI:** 10.4076/1757-1626-2-7150

**Published:** 2009-09-11

**Authors:** Dibendu Betal, Jasdeep S Khangura, Peter J Swan, Veysi Mehmet

**Affiliations:** 1 Department of Surgery, St Mary's Hospital, Isle of Wight NHS Trust, Parkhurst Road, Newport, Isle of Wight, PO30 5TG, UK

## Abstract

Splenic artery aneurysms are rare. We discuss a case of a 58-year-old gentleman presenting with collapse and shock secondary to spontaneous splenic artery aneurysm rupture. Patient underwent laparotomy and splenectomy then discharged home within a week of presentation.

## Introduction

Splenic artery aneurysms are a rare clinical diagnosis. They may either be due to congenital defects or acquired conditions for example secondary to atherosclerosis formation. They are more common in women especially in the third trimester of pregnancy. Clinically they may present with epigastric pain or may be asymptomatic. Initial bleeding may be localised to the lesser sac followed by free intraperitoneal haemorrhage then shock and collapse. Treatment for ruptured splenic artery aneurysm with shock is immediate surgery, resection of aneurysm with or without splenectomy.

## Case presentation

A 58-year-old White British gentleman was admitted to Accident and Emergency with a sudden onset of epigastric and lower chest pain and collapse. On arrival to A&E the patient was clammy, pale and agitated. He was previously fit and healthy with a left hernia repair his only previous admission to hospital.

**Figure 1:**
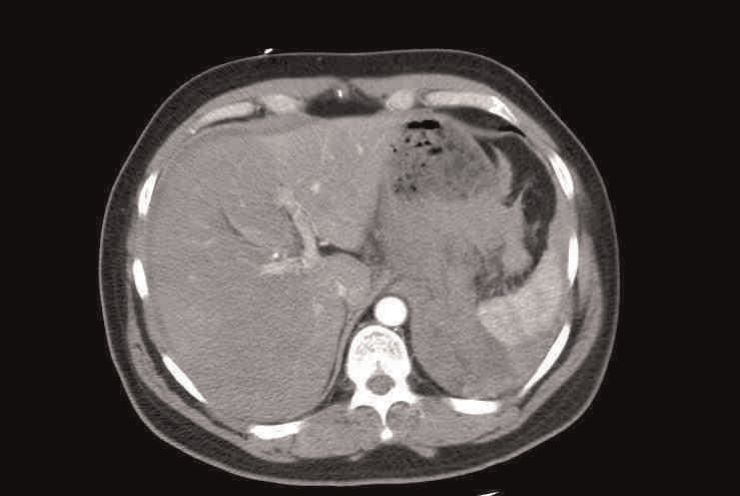
**Intraperitoneal haemorrhage secondary to splenic artery aneurysm rupture**.

His observations demonstrated hypovolaemic shock with hypotension (68/48 mmHg), tachycardia (115 bpm) and tachypnoea (rate 27/min). His abdomen was rigid with generalised tenderness. Blood gasses showed metabolic acidosis with a slightly lowered haemoglobin (11 g/dl). ECG showed depressed ST waves in the lateral leads indicating ischaemic event. The provisional diagnosis was either a thoracic or abdominal aortic aneurysm rupture. Patient underwent a CT scan that demonstrated large intraperitoneal haemorrhage (Figure 1). The splenic artery was dilated compared to the celiac axis and was tortuous and irregular. There was no other pathology seen.

Patient was taken to theatre for a laparotomy - intra-operative findings showed nearly two litres of intra-abdominal blood in the lesser sac behind the pancreas secondary to splenic artery aneurysm. Splenic pedicle and vein were ligated and splenectomy was performed. Histological examination of the specimen confirmed the diagnosis.

The patient had a four-day stay in the Intensive Care Unit then a further two more days on a general surgical ward before discharge. Patient had inoculation for splenectomy and started on penicillin then discharged from surgical clinic six weeks after initial presentation.

## Discussion

Splenic artery aneurysms are rare aneurysms compared to aortic and iliac artery aneurysm. They are often found incidentally on post-mortem examination or imaging of the upper abdomen. Main complication is rupture leading to massive intraperitoneal haemorrhage. Incidence has been reported to be between 0.02% and 0.1%, it is found in all age groups with a peak in fifth and sixth decades of life and more frequently in women [1].

The cause of splenic artery aneurysms may be from portal hypertension secondary to liver cirrhosis [2], atherosclerosis [3] and pregnancy [4]. It is not fully understood why pregnancy is a cause but it may be due to multiparity and associated hormonal effect and portal hypertension causing dilatation of the aneurysm.

The earlier case reports present the rupture of splenic artery aneurysm during pregnancy [5]. An initial review of the literature suggests that the maternal mortality rate is 72% and foetal mortality of 97% [6]. There is a reported case of post-partum splenic artery aneurysm rupture [7]. Rupture tends to occur in the final trimester of pregnancy and prolonged hypertension may be a contributory factor.

Other causes of ruptured splenic artery aneurysms include congenital causes such as anomalous origin [8], Berry aneurysm [9] and arteriovenous malformation [10] and acquired conditions such as pancreatitis [11] and possible toxic causes resulting in vessel wall damage [12].

Presentation may be asymptomatic or presenting with severe epigastric pain with or without shock. Prior to shock there is often intra-abdominal haemorrhage. There have been reports of haemorrhage into stomach [13], colon [14] and pancreas [15].

The traditional treatment of ruptured splenic artery rupture is open surgery, aneurosectomy with or without splenectomy [16]. Recent advances include the popular use of laparoscopic surgery [17] and non-operative endovascular management of splenic artery aneurysms [18].

The risk of splenic artery aneurysm rupture ranges between 3-10%, with a mortality of 25%. If the aneurysm is treated before rupture the mortality rate is less than 0.5%. Mortality rate after surgical treatment is less than 25% [19]-[20].

## Conclusion

Splenic artery aneurysms are a rare form of aneurysm and are often asymptomatic. However it is a diagnosis to consider when a patient presents with severe epigastric pain and shock especially in pregnancy.

## Consent

Written informed consent was obtained from the patient for publication of this case report and any accompanying images. A copy of the written consent is available for review by the Editor-in-Chief of this journal.

## Competing interests

The authors declare that they have no competing interests.

## Authors' contributions

DB involved in writing the paper, JSK did literature search, PJS performed surgical procedure, VM performed surgical procedure and final revision of article.
